# TACE-Sorafenib With Thermal Ablation Has Survival Benefits in Patients With Huge Unresectable Hepatocellular Carcinoma

**DOI:** 10.3389/fphar.2020.01130

**Published:** 2020-07-29

**Authors:** Ying Wu, Han Qi, Fei Cao, Lujun Shen, Shuanggang Chen, Lin Xie, Tao Huang, Ze Song, Danyang Zhou, Weijun Fan

**Affiliations:** ^1^ Department of Minimally Invasive Interventional Therapy, Sun Yat-sen University Cancer Center, Guangzhou, China; ^2^ Department of Medical Oncology, Sun Yat-sen University Cancer Center, Guangzhou, China; ^3^ State Key Laboratory of Oncology in South China, Collaborative Innovation Center of Cancer Medicine, Sun Yat-sen University, Guangzhou, China

**Keywords:** hepatocellular carcinoma, transarterial chemoembolization, thermal ablation, sorafenib, treatment, drug resistance

## Abstract

**Purpose:**

To investigate the effectiveness and safety of transarterial chemoembolization (TACE) combined with sorafenib and thermal ablation in patients with huge hepatocellular carcinoma (HCC).

**Materials and Methods:**

This retrospective study examined 50 patients with huge unresectable HCC treated from January 2009 to December 2015. Among them, 28 cases received TACE-sorafenib treatment (TACE-sorafenib group), and 22 cases received TACE-sorafenib plus thermal ablation treatment (TACE-sorafenib-thermal ablation group). The Overall survival (OS), progression-free survival (PFS), and adverse events (AEs) were compared.

**Results:**

The median follow-up was 13.5 months (ranges 4.2 to 96.7 months). The median OS was significantly longer in the TACE-sorafenib-thermal ablation group than that in the TACE-sorafenib group (20.8 vs. 10.4 months, *P*=0.003). The median PFS of the ablation and no ablation groups were 4.3 vs. 7.1 months (*P*=0.546). The treatment modality was an independent predictor of OS (*P*=0.004). There were no notable drug-related high grade adverse events or permanent adverse sequelae.

**Conclusion:**

TACE-sorafenib-thermal ablation provided extended OS to patients with huge unresectable HCC and could be a better choice than TACE-sorafenib.

## Introduction

Hepatocellular carcinoma (HCC) is the second most common cause of cancer-related mortality in China (Jemal et al., 2011). Huge unresectable HCC (i.e., >10 cm in its larger axis) is encountered in a considerable portion of patients at diagnosis.

Huge HCCs have specific features that need to be taken into account for successful management. Firstly, HBV-related liver cirrhosis is the predominant underlying cause of HCC in China (Chen et al., 2016). Secondly, huge HCCs always show an incomplete capsule and are prone to invade local vasculature, increasing the risk of tumor thrombus and metastasis (Xue et al., 2015). Thirdly, huge HCCs have an increased risk of rupture, which may accelerate their local spread and deterioration of liver function (Poon et al., 2002). Hence, adequate management of huge HCCs is a challenge.

For unresectable huge HCC, multiple approaches such as sorafenib adminstration, transarterial chemoembolization (TACE), and thermal ablation can be used for its management. Sorafenib, is approved in patients with unresectable HCC based on two phase III randomized trials (Llovet et al., 2008; Cheng et al., 2009) and is the recommended treatment for patients with advanced HCC (Omata et al., 2010; European Association For The Study Of The et al., 2012). However, the efficacy of sorafenib monotherapy is generally limited. Sorafenib is beneficial in only approximately 30% of patients, and acquired resistance often develops within 6 months, with a mean overall survival (OS) and time to progression of 10.7 and 5.5 months, respectively, in the SHARP study (Cheng et al., 2009), and 6.5 and 2.8 months, respectively, in the Asia-Pacific study (Llovet et al., 2008).

TACE is considered as the standard treatment for unresectable HCC (Llovet et al., 2002; Llovet and Bruix, 2003), but TACE monotherapy rarely results in complete necrosis of the lesions (Han et al., 2019). In addition, TACE increases VEGF levels, and VEGF is known to stimulate tumor angiogenesis, thereby contributing to tumor invasion and metastasis (Sergio et al., 2008; Shim et al., 2008). Thermal ablation therapies such as radiofrequency ablation (RFA) and microwave ablation (MWA) have been shown to be safe and effective for local control in patients with HCC (Lee et al., 2014; Hernandez et al., 2015). For early-stage HCC, thermal ablation has been shown to have similar OS compared with surgical resection (Lee et al., 2014; Hernandez et al., 2015). For moderate to advanced stage HCC, thermal ablation can provide a good local control (Lee et al., 2014; Hernandez et al., 2015). Both TACE and thermal ablation are local therapies, and they have limited preventive effect against tumor recurrence and metastasis.

In recent years, multimodal approaches are recommended in unresectable HCC, either as first-line or subsequent therapy. Some studies have shown that the combination of TACE and sorafenib or TACE and thermal ablation (Cabrera et al., 2011; Kim et al., 2011; Kudo et al., 2011) is superior to monotherapy. However, few data are available on patients with huge unresectable HCCs treated by TACE and sorafenib, with or without thermal ablation. In the present study, we retrospectively evaluate and compare the benefits of TACE and sorafenib with or without thermal ablation in the management of patients with huge unresectable HCCs.

## Materials and Methods

### Patients

This study followed the requirements of the Declaration of Helsinki and was approved by the Institutional Review Board of Sun Yat-sen University Cancer Center. This single-center retrospective study examined the clinical data of patients with huge unresectable HCC treated from January 2009 to December 2015 at Sun Yat-sen University Cancer Center.

The inclusion criteria were: (1) diagnosis of HCC confirmed by liver biopsy or clinically according to the American Association for the Study of Liver Diseases (AASLD) criteria (Llovet et al., 2008); (2) original tumor ≥10 cm in diameter, and satellite foci ≤2 cm; (3) All target lesions that could be measured according to the modified Response Evaluation Criteria in Solid Tumors Group (mRECIST) guidelines (Lencioni and Llovet, 2010); (4) 18–80 years of age; and (5) underwent TACE with sorafenib or TACE, sorafenib, and thermal ablation treatment. The exclusion criteria were: (1) procedure other than TACE and thermal ablation; (2) extrahepatic HCC metastases; (3) portal vein tumor thrombus beyond type IIa (Chan et al., 2016); or (4) previous treatment for HCC.

A total of 533 patients with HCC were treated with sorafenib in our cancer center during the study period. According to the inclusion/exclusion criteria, only 50 patients with huge HCC were included in the present analysis.

### Treatment Option

Prior to their treatment, the patients were fully informed of the specific implications of TACE-sorafenib and TACE-sorafenib-thermal ablation therapies, as well as the possible adverse effects (AEs). The fact that there is limited evidence on the treatment effect was emphasized. Then, the patient received the treatment he/she selected. Informed treatment consent was obtained from all patients before treatment.

### Transarterial Chemoembolization

All TACE treatments were performed by three physicians who have at least 5 years of experience. Under local anesthesia, a 5F French catheter (Yashiro type; Terumo Corporation, Tokyo, Japan) was introduced into the abdominal aorta *via* the femoral artery using the Seldinger technique. Hepatic arterial angiography was performed using fluoroscopy to guide the catheter into the celiac and superior mesenteric arteries. The feeding arteries, tumor, and vascular anatomy surrounding the tumor were identified. A microcatheter (Renegade Hi Flo; Boston Scientific Corporation, Boston MA, USA) was super-selectively inserted into the feeding arteries. A solution containing pirarubicin (10–40 mg, Shenzhen Main Luck Pharmaceuticals Inc., China), lobaplatin (50 mg/m^2^, Hainan Changan International Pharmaceutical Co., Ltd., China), and 10 mg of mitomycin c (Zhejiang Hisun pharmaceutical Co., Ltd., China) in iodized oil (Lipoid ultra-fluid, Guerbet, France) was infused into the arteries according to the number and size of the lesions. Liver and kidney function of the patient, and blood supply of the tumors (Chung et al., 2011) were evaluated. Follow-up imaging was performed 1–3 months later to evaluate the effects of TACE. The treatment was repeated once every 1–2 months. Reexamination was conducted one month after TACE. If there were still enhanced tumors, repeated thermal ablation therapy was required. Treatments were discontinued in the presence of significant liver function deterioration or complete elimination of the liver tumors.

### Thermal Ablation

All thermal ablation treatments were performed by three physicians who have at least 5 years of experience. For patients who were willing to receive thermal ablation therapy, we usually performed 3–4 TACE sessions for hypervascular HCCs and 2–3 TACE sessions for hypovascular HCCs before performing thermal ablation based on our experience. Patients were instructed to fast from all foods for 12 h before the procedure. Computed tomography (CT) (Brilliance CT Big Bore; Philips, Best, The Netherlands) was used to locate the liver tumors and to design the optimal route for puncture needle. Routine disinfection and local anesthesia were applied around the puncture points. A microwave antenna (FORSEA; Qinghai Microwave Electronic Institute, Nanjing, China) or RFA (RITA2000, Mountain View. California, USA) was gradually inserted into the tumor along the predetermined angle. The whole MWA procedure was conducted under intravenous anesthesia (propofol; AstraZeneca, London, UK). Vital signs were monitored during the procedure. The settings of the ablation parameters depended upon the manufacturer’s recommendation and our experience. An upper abdominal CT scan was carried out immediately after the procedure to evaluate the ablation area and complications. Routine blood and biochemical tests were performed on the first day after ablation treatment to monitor for eventual complications. After the first thermal ablation, TACE was repeated according to the proportion of residual tumor or disease status. If the proportion of residual tumor was >50% or new tumors appeared in the liver, additional TACE were performed.

### Sorafenib Treatment

All patients received continuous standard doses of sorafenib (400 mg twice a day, orally). Sorafenib was started from 3 days to 2 months after the TACE treatment or when tumor progression was found. During sorafenib treatment, the patients visited the outpatient clinic every three or four weeks for AE and tolerability assessments. Dose adjustments were made based on clinically significant toxicity (grade 3 or 4 according to the National Cancer Institute Common Terminology Criteria for Adverse Events Version 4.0) or the determination of patient tolerance by clinicians. For patients who have grades 3 and 4 toxicities, sorafenib was withdrawn until the symptoms improved to grade 2 or lower. Sorafenib was reintroduced at a dose of 200 mg bid for 5 days and then increased back to 400 mg bid if well tolerated. Otherwise, sorafenib was continued at 200-mg bid. Sorafenib was continued until toxicities were unmanageable.

### Follow-Up

OS was calculated from the date of treatment of HCC until the date of the final follow-up or death (no patient was lost in follow-up). Progression-free survival (PFS) was calculated from the day of diagnosis to radiologic progression based on the modified Response Evaluation Criteria in Solid Tumors (mRECIST) evaluation (Lencioni and Llovet, 2010). The procedure-related complications of TACE and thermal ablation were evaluated based on the guidelines for trans-catheter therapy and image-guided tumor ablation (Brown et al., 2009; Goldberg et al., 2009). Major complications were defined as events that led to substantial morbidity or disability, required hospital admission, or substantially lengthened hospital stay (Sacks et al., 2003). All other complications were considered minor. The drug-related toxicity was observed and recorded according to the National Cancer Institute Common Terminology Criteria for Adverse Events Version 4.0.

Liver function, blood coagulation profile, and serum alpha-fetoprotein (AFP) levels were examined monthly. A three-phase helical CT (HiSpeed or LightSpeed QX/I; GE Medical Systems, Milwaukee, WI, USA) or MRI (Discovery MR750 3.0T; GE Medical Systems, Milwaukee, WI, USA) examination was carried out every month for the first 3 months post-operatively. Patients with residual tumor were re-treated with the original procedure. If no residual tumor or tumor recurrence was found, re-examinations were carried out every 3–6 months. Follow-up was censored on June 30, 2017.

### Statistical Analysis

Continuous variables were presented as mean ± standard deviation (SD) and analyzed using the Student’s *t* test. Categorical variables were analyzed using the Chi-square or Fisher’s exact test, as appropriate. Survival rates were estimated by the Kaplan-Meier method. Differences in OS were assessed for significance using the log-rank test. The Cox proportional hazards regression model was used to determine the factors associated with survival. As per initial design, all variables with a *P*<0.05 by univariable analysis were entered in the multivariable analysis; finally, only one variable was found to be associated with survival and multivariable analysis could not be performed. All analyses were performed using SPSS 13 (SPSS, Inc., Chicago, IL, USA). Two-tailed *P*-values <0.05 were considered statistically significant.

## Results

### Baseline Clinical Characteristics

Among 533 patients with HCC who were treated with sorafenib at our hospital between January 2009 and December 2015, 28 and 22 patients with huge HCCs were treated with TACE-sorafenib and TACE-sorafenib-thermal ablation therapy, respectively. The baseline characteristics of these patients are shown in **Table 1**. In the TACE-sorafenib group, the 28 patients with 56 liver tumors received 72 TACE treatments (2.6 ± 1.3, range: 1–7). In the TACE-sorafenib-thermal ablation group, 22 patients with 40 liver tumors received 88 TACE treatments and 71 ablations. The specific information about RFA and MWA procedures are provided in **Table 2**.

**Table 1:** Baseline characteristics of the patients.

Variables	TACE-sorafenib-thermal ablation (n=22)	TACE-sorafenib (n=28)	*P*
**Sex**			>0.999*
Female	0	1 (3.6%)	
Male	22 (100%)	27 (96.4%)	
**Age (years)**	46.4 ± 11.9	48.8 ± 12.3	0.479
<50	15 (68.2%)	15 (53.6%)	0.295
≥50	7 (31.8%)	13 (46.4%)	
**HBV infection**			>0.999*
Positive	22 (100%)	27 (96.4%)	
Negative	0	1 (3.6%)	
**AFP (ng/ml)**			0.522
<400	9 (40.9%)	9 (32.1%)	
≥400	13 (59.1%)	19 (67.9%)	
**Child-Pugh grade**			0.246*
A	22 (100%)	25 (89.3%)	
B	0 (0%)	3 (10.7%)	
**BCLC stage**			0.749*
A	4 (18.2%)	3 (10.7%)	
B	6 (27.3%)	8 (28.6%)	
C	12 (54.5%)	17 (60.7%)	
**Tumor diameter (cm)**	12.3 ± 2.5	12.3 ± 2.4	0.988
**Tumor number**	1.82 ± 0.8	1.96 ± 1.0	0.559
1	9 (40.9%)	13 (46.4%)	0.696
2–3	13 (59.1%)	15 (53.6%)	
**Growth pattern**			0.481*
Infiltrative	5 (22.7%)	4 (14.3%)	
Noninfiltrative	17 (77.3%)	24 (85.7%)	
**Vascular invasion**			0.449
Present	11 (50.0%)	11 (39.3%)	
Absent	11 (50.0%)	17 (60.7%)	

*The Fisher exact test was used.

**Table 2:** Procedure-related information about radiofrequency ablation (RFA) and microwave ablation (MWA) in the transarterial chemoembolization (TACE)-sorafenib-thermal ablation group.

Variables	TACE-sorafenib-thermal ablation		*P*
	RFA (n=11)	MWA (n=9)	
**Number of procedures**			0.702
Range	1–7	2–5	
Mean ± SD	2.8 ± 1.9	3.1 ± 1.3	
**Procedure duration (min)**			0.385
Range	8–110	6–118	
Mean ± SD	45.6 ± 31.4	39.0 ± 25.3	
**Number of ablation sites**			0.306
Range	1–7	1–8	
Mean ± SD	3.4 ± 1.8	3.9 ± 2.0	
**Ablation duration at each site (min)**			<0.01
Range	5–25	3–20	
Mean ± SD	13.2 ± 4.7	9.0 ± 3.5	
**Hospital stays (days)**			<0.01
Range	1–7	1–5	
Mean ± SD	3.48 ± 1.1	2.46 ± 0.9	

### Patient Survival and Tumor Progression

**Figure 1:**
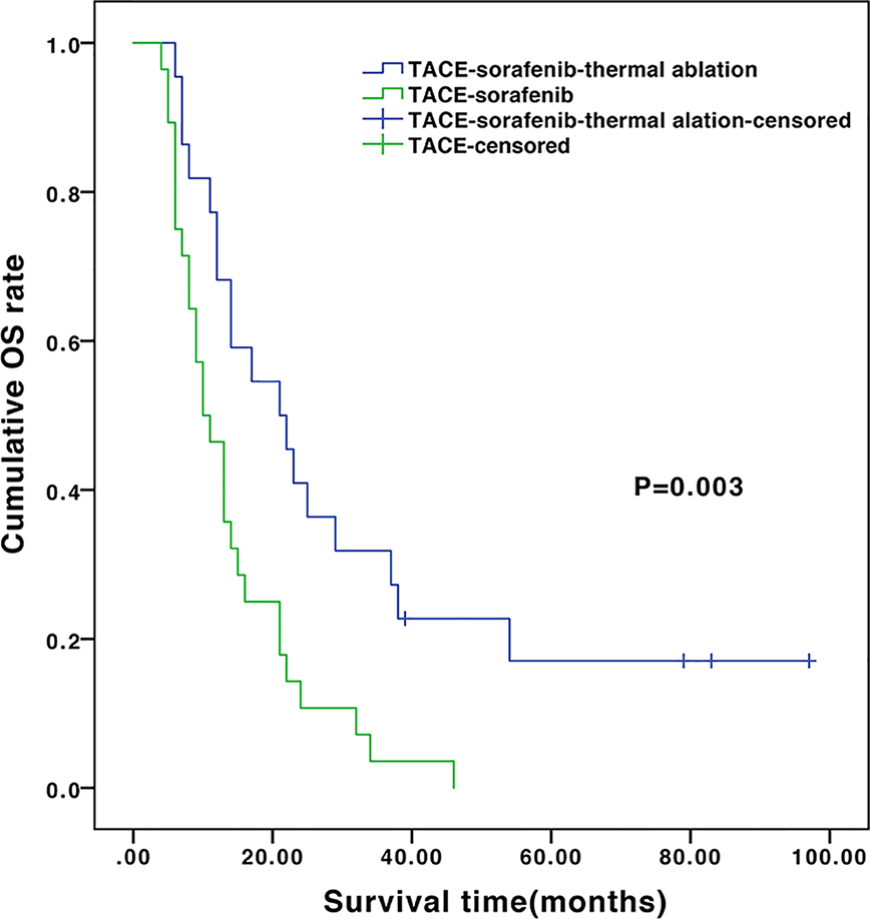
Overall survival of the transarterial chemoembolization (TACE)‑sorafenib-thermal ablation and TACE-sorafenib groups.

**Figure 2:**
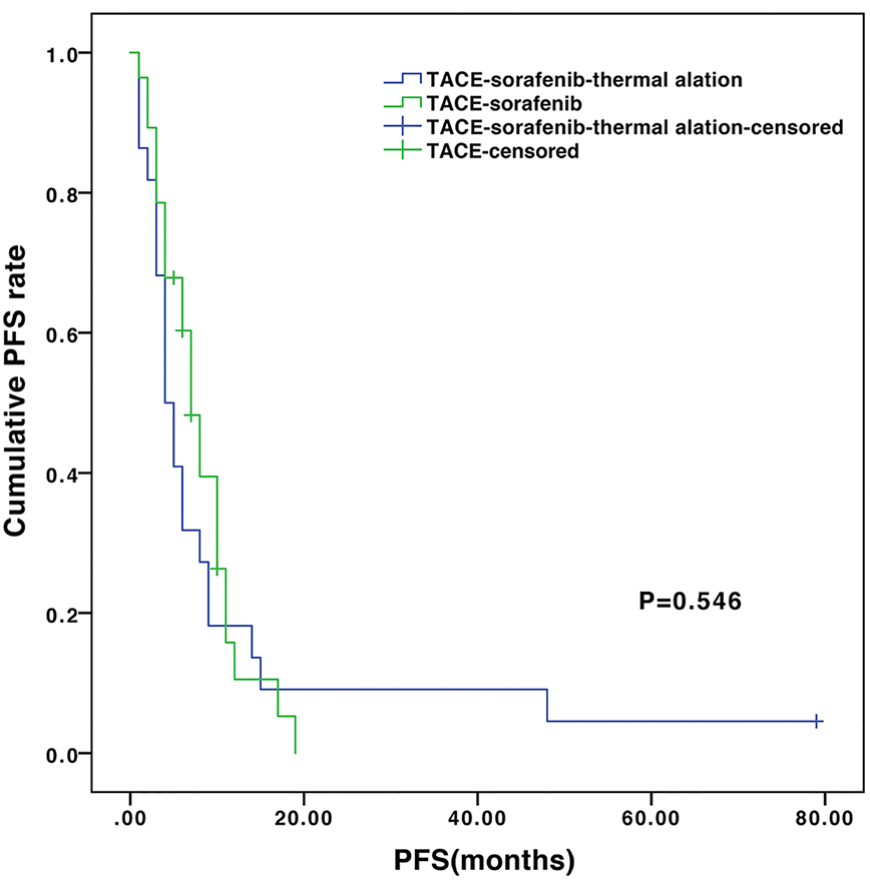
Progression-free survival of the transarterial chemoembolization (TACE)-sorafenib-thermal ablation and TACE-sorafenib groups.

The median follow-up of the entire cohort was 13.5 months (ranges 4.2–96.7 months). During follow-up, 28 (100%) and 18 (81.8%) patients died in the TACE-sorafenib and TACE-sorafenib-thermal ablation group, respectively. The median OS was significantly longer in the TACE-sorafenib-thermal ablation group than in the TACE-sorafenib group (20.8 vs. 10.4 months, *P*=0.003) (**Figure 1**). The 1-, 2-, and 3-year cumulative survival rates in the TACE-sorafenib-thermal ablation and TACE-sorafenib groups were 68.2%, 40.9%, and 31.8% vs. 46.4%, 10.7%, and 3.6%, respectively (all *P*<0.05). The median PFS of the TACE-sorafenib-thermal ablation and TACE-sorafenib groups were 4.3 vs. 7.1 months, respectively (*P*=0.546) (**Figure 2**).

**Figure 3:**
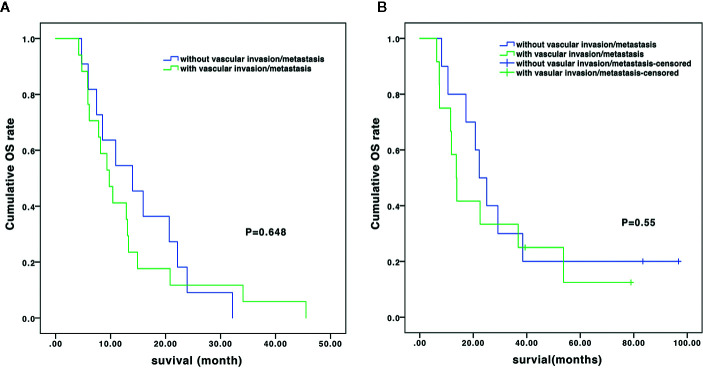
Overall survival of patients with and without vascular invasion/metastasis in the transarterial chemoembolization (TACE)-sorafenib group **(A)** and the TACE-sorafenib-thermal ablation group **(B)**.

**Figure 4:**
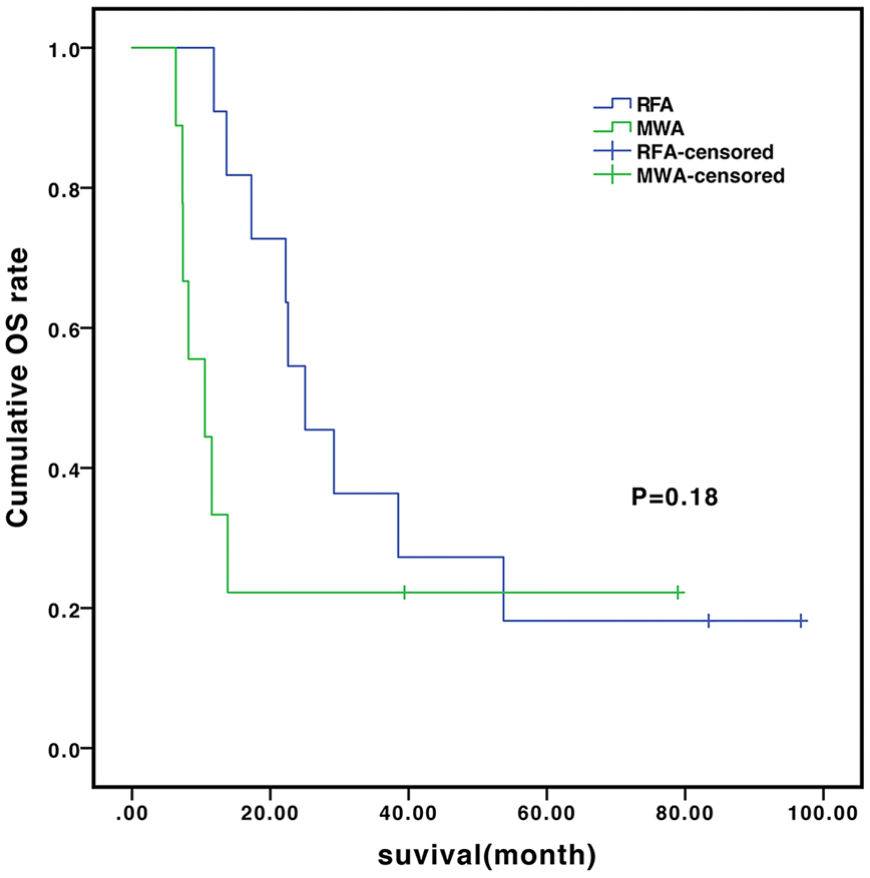
Overall survival of patients who received radiofrequency ablation (RFA) and microwave ablation (MWA) in the transarterial chemoembolization (TACE)-sorafenib-thermal ablation group.

In the subgroup analysis, the median OS of the patients without and with vascular invasion/metastasis were 14.0 vs. 9.8 months (*P*=0.648) in the TACE-sorafenib group, and 22.2 vs. 13.7 months (*P*=0.55) in the TACE-sorafenib-thermal group (**Figure 3**). The median OS of the patients who received RFA and MWA alone were 25.0 vs. 10.5 months (*P*=0.18) in the TACE-sorafenib-thermal ablation group (**Figure 4**).

### Cox Analysis

The predictors of OS in the Cox analysis are shown in **Table 3**. Multivariate analysis was not performed because only the choice of treatment had a *P*-value <0.05 in univariable analyses. HBV status and Child-Pugh grades could not be analyzed because the number of patients in some categories are too small. TACE-sorafenib-thermal ablation (HR=2.512, 95%CI:1.348–4.680, *P*=0.004) was found to be a predictor of OS.

**Table 3:** Factors influencing overall survival according to the Cox analysis.

Variables	HR (95% CI)	*P*
**AFP (ng/ml)**		
<400	1.000	
≥400	1.317 (0.772–2.401)	0.369
**BCLC stage**		
A	1.000	
B	2.097 (0.791–5.558)	0.137
C	2.108 (0.865–5.137)	0.101
**Tumor number**		
Single	1.000	
Multiple	1.385 (0.768-2.498)	0.279
**Growth pattern**		
Non-infiltrative	1.000	
Infiltrative	1.555 (0.743–3.255)	0.241
**Vascular invasion**		
Absent	1.000	
Present	1.259 (0.701–2.262)	0.441
**Therapy**		
TACE-sorafenib-thermal ablation	1.000	
TACE-sorafenib	2.512 (1.348–4.680)	0.004

Multivariate analysis was not performed because only the choice of treatment had a P-value <0.05 in univariable analyses. HBV status and Child-Pugh grades could not be analyzed because of the too small number of patients in some categories.

### Adverse Events

The most common drug-related toxicities were hand-foot syndrome (HFS, 86.4% vs. 85.7%), alopecia (31.8% vs. 39.3%), diarrhea (18.2% vs. 35.7%), and hypertension (13.6% vs. 3.6%) in the TACE-sorafenib-thermal ablation and TACE-sorafenib groups, respectively. Most adverse events were grade 1 or 2. Four cases of drug-related grade 3 toxicitiy were reported, including one case of HFS in the TACE-sorafenib-thermal ablation group and three cases (one with HFS, one with bleeding, and one with liver dysfunction) in the TACE-sorafenib group. No drug-related grade 4–5 AEs were recorded. All drug-related toxicities are listed in **Table 4**.

**Table 4:** Drug-related toxicity.

Adverse event	TACE-sorafenib-ablation (n=22)		TACE-sorafenib (n=28)	
	Any grade	Grade 3	Any grade	Grade 3
Hand-foot reaction	19 (86.4%)	1 (4.5%)	24 (85.7%)	1 (3.6%)
Rash	8 (36.4%)	0	6 (21.4%)	0
Diarrhea	4 (18.2%)	0	10 (35.7%)	0
Hypertension	3 (13.6%)	0	1 (3.6%)	0
Voice changes	1 (4.5%)	0	2 (7.1%)	0
Bleeding	0	0	1 (3.6%)	1 (3.6%)
Liver dysfunction	0	0	1 (3.6%)	1 (3.6%)
Alopecia	7 (31.8%)	0	11(39.3%)	0

The most common minor complications were post-embolization/ablation syndrome which includes fever (37.7% vs. 83.3%), pain (28.9% vs. 15.3%), and vomiting (7.5% vs. 4.2%) in the TACE-sorafenib-thermal ablation and TACE-sorafenib group, respectively. Meanwhile, two cases of minor bleeding (one thoracic hemorrhage and one liver subcapsule hemorrhage) were observed in the TACE-sorafenib-thermal ablation group; both cases resolved without special treatment. Four major complications were reported, including two cases of liver dysfunction in the TACE-sorafenib-thermal ablation group and two cases of myelosuppression in the TACE-sorafenib group. No permanent adverse sequelae or treatment-related death were observed. All procedure-related complications are listed in **Table 5**.

**Table 5:** Procedure-related complications.

Procedure-related complications	TACE-sorafenib-thermal ablation	TACE-sorafenib
	(n=159)	(n=72)
**Minor complications**		
**Postembolization/ablation syndrome**		
Fever	60 (37.7%)	60 (83.3%)
Pain	46 (28.9%)	11 (15.3%)
Vomiting	12 (7.5%)	3 (4.2%)
**Bleeding**		
Thoracic hemorrhage	1 (0.6%)	0
Liver subcapsule hemorrhage	1 (0.6%)	0
**Major complications**		
**Liver dysfunction**	2 (1.3%)	0
**Myelosuppression**	0	2 (2.8%)

## Discussion

Few data are available on the treatment strategies for huge HCC. This study compares the effectiveness and adverse events (AEs) of TACE combined with sorafenib and with or without thermal ablation in patients with huge unresectable HCC. The results suggest that TACE-sorafenib-thermal ablation provided an extended long-term OS to patients with huge unresectable HCC. TACE-sorafenib-thermal ablation may be a better choice than TACE-sorafenib for huge unresectable HCC.

TACE-sorafenib therapy has received more and more acceptance for the treatment of HCC. Compared with the studies of TACE monotherapy for huge HCCs by Xue et al. (2015) and Min et al. (2014), the present study suggests that the TACE-sorafenib group had a higher 1-year survival rate than the studies by Xue et al. (2015) and Min et al. (2014) (46.4% vs. 33% and 37.8%), despite the fact that the patients in the present study had a high risk of vessel invasion and multiple tumors. Hence, TACE-sorafenib therapy could provide survival benefits for patients with huge unresectable HCC. However, TACE is not a curative treatment and the rates of objective response range from 16% to 60% (Llovet and Bruix, 2003), suggesting limited benefits for huge HCC, particularly for hypovascular HCC. In addition, post-TACE vascular changes and hepatic dysfunction caused by sequential TACEs ultimately limit the number of TACE treatments that a patient can receive. Thus, TACE-sorafenib could only provide limited survival benefits for huge unresectable HCC.

Thermal ablation is a minimally invasive technique and is increasingly being used in managing HCCs. The present study provided evidence that thermal ablation plus TACE and sorafenib could be used for the treatment of huge unresectable HCC. In the present study, patients in the TACE-sorafenib-thermal ablation group had significantly higher 1-, 2-, and 3-year survival rates and longer median survival than those in the TACE-sorafenib group. Moreover, although all patients included in the present study had unresectable huge HCCs, Mok et al. (2003) and Hwang et al. (2015) reported that patients treated with TACE+MWA had a similar 1-year OS survival rate (68.6%) compared with surgical resection. TACE-sorafenib-thermal ablation has the following advantages. Firstly, increased levels of proangiogenic factors following thermal ablation have been reported and may be a potential reason for tumor recurrence (Dong et al., 2015). As reported by Dong et al. (2015), sorafenib inhibits the up-regulation of p-Akt and p-ERK1/2 in HCC cells after insufficient RFA, and further down-regulates the expression of N-cadherin, vimentin, and snail, resulting in enhanced migration and invasion of HCC cells after insufficient RFA. Secondly, thermal ablation can directly and efficiently kill tumor cells and lighten tumor load, especially for hypovascular HCC or HCC with arteriovenous fistula, in which failures to TACE are common. Thermal ablation can specifically eliminate residual tumor tissues after TACE, which could increase the local control rate and reduce neovascularization. Thirdly, thermal ablation causes little damage on liver function and allows an extension of the time interval between two TACE treatments, thus protects liver function. Finally, thermal ablation, especially RFA, could enhance various TAA-specific T cell response and release tumor antigen, heat shock protein, etc., thereby improving tumor-specific immunity, killing residual tumor cells, and reducing recurrence (Mizukoshi et al., 2013). Therefore, TACE-sorafenib-thermal ablation therapy could have great benefits for the treatment of huge unresectable HCC.

In the present study, TACE-sorafenib therapy was the only independent predictor for poor prognosis according to the Cox analysis, suggesting that the addition of thermal ablation is of vital importance in treating huge unresectable HCC. In the ablation procedure, the following principles are taken into account at our hospital and may contribute to the survival benefits. Firstly, for huge HCCs with satellite foci, the original or relatively larger tumor is always the first MWA target. Secondly, MWA should be performed first in the center of the tumor, then in the peripheral residual lesions. Finally, MWA ablation should be given to tumor located in a relatively safe place, then in the tumor that is adjacent to susceptible organs such as the intestines and gallbladder.

The difference of PFS between the TACE-sorafenib-thermal ablation and TACE-sorafenib groups was not statistically significant. We noticed that the median PFS and the 1-year PFS rates were longer in the TACE-sorafenib group, which may be caused by different sorafenib treatment time. The patients in the TACE-sorafenib-thermal ablation group began to take sorafenib at a later time than the patients in the TACE-sorafenib group. Kudo et al. (2011) reported that sorafenib did not significantly prolong survival in patients who responded well to TACE, due to delays in starting sorafenib (>9 weeks) after TACE. Therefore, we recommend starting sorafenib as soon as possible.

In the subgroup analysis, as in the study by Qu et al. (2012), we observed that there was no significant difference in OS between patients with and without vascular invasion in the two groups. Meanwhile, there was no significant difference in OS between patients who received only MWA and RFA treatment in the TACE-sorafenib-thermal ablation group. Nevertheless, patients who received MWA treatment had a slightly shorter time of ablation per site, and this may be due to its advantages over RFA, i.e., more rapid heating rate, higher intratumor temperature (reaching 130°C), larger ablation range, deeper tissue penetration, and smaller influence of blood flow (Hernandez et al., 2015). Hence, it is suggested that priority should be given to MWA for the treatment of huge unresectable HCCs, but this has to be validated by future studies.

In terms of AEs, the present study suggests that TACE-sorafenib-thermal ablation was well tolerated and led to only manageable side effects in patients with huge unresectable HCC. The most common reported drug-related toxicities were HFS, alopecia, diarrhea, and hypertension, similar to previous studies (Abdel-Rahman and Elsayed, 2013). Furthermore, TACE-sorafenib-thermal ablation did not increase the procedure-related complications in patients with huge unresectable HCC. The most common reported procedure-related complications were post-embolization/ablation syndrome, similar to a study by Yao et al. (2015). The major complications in the TACE-sorafenib-thermal ablation and TACE-sorafenib groups were liver dysfunction (n=2) and myelosuppression (n=2), similar to the 4.6% incidence reported for large HCCs by previous studies (Paul et al., 2011; Liang et al., 2013), but lower than in another study by Dong et al. (2016), which could be due to the choice of drugs for TACE. Meanwhile, no permanent adverse sequelae or treatment-related death were observed. Thus, these results suggest that TACE-sorafenib-thermal and TACE-sorafenib are well tolerated by patients with huge unresectable HCC.

There are some limitations in the current study. Firstly, it was a retrospective study and treatment strategy was determined by the choice of patients. Secondly, it was a single-center study in China. The HBV infection rate was higher in China than in Western countries. Thirdly, the sample size might not be large enough. In addition, our study failed to reveal the difference of RFA and MWA and risks factors for prognosis, maybe due to the relatively small sample size. Fourthly, the duration of sorafenib use in these two groups was not the same, which may have affected the OS. Therefore, a prospective trial with a larger sample size is needed.

In conclusion, the present study provides the evidence that TACE-sorafenib and TACE-sorafenib-thermal ablation are well-tolerated and beneficial in patients with huge unresectable HCC. TACE-sorafenib-thermal ablation is associated with significant survival benefits for patients with huge unresectable HCCs compared with TACE-sorafenib.

## Data Availability Statement 

The raw data supporting the conclusions of this article will be made available by the authors, without undue reservation.

## Ethics Statement

This study met the requirements of the Declaration of Helsinki and was approved by the Institutional Review Board of Sun Yat-sen University Cancer Center.

## Author Contributions

WF: Study conception. YW, HQ, FC: Analysis and interpretation of data. All authors contributed to the article and approved the submitted version.

## Funding

This work was funded by the National Natural Science Foundation of China. Grant number: 81771954.

## Conflict of Interest

The authors declare that the research was conducted in the absence of any commercial or financial relationships that could be construed as a potential conflict of interest.

## Abbreviations

AEs, adverse events; AFP, alpha-fetoprotein; CT, computed tomography; HCC, hepatocellular carcinoma; mRECIST, modified Response Evaluation Criteria in Solid Tumors; MWA, microwave ablation; OS, overall survival; PFS, progression-free survival; RFA, radiofrequency ablation; TACE, transarterial chemoembolization.
